# COVID-19 and indirect health implications in Africa: Impact, mitigation measures, and lessons learned for improved disease control

**DOI:** 10.1371/journal.pmed.1003666

**Published:** 2021-06-23

**Authors:** Seth C. Inzaule, Pascale Ondoa, Marguerite Massinga Loembe, Yenew Kebede Tebeje, Ahmed E Ogwell Ouma, John N. Nkengasong

**Affiliations:** 1 Africa Centres for Disease Control and Prevention, African Union Headquarters, Addis Ababa, Ethiopia; 2 African Society for Laboratory Medicine, Addis Ababa, Ethiopia

## Abstract

Seth Inzaule and co-authors discuss implications of the COVID-19 pandemic for health in African countries.

**Summary points**

- Coronavirus Disease 2019 (COVID-19) and the mitigation measures taken to limit its spread have significantly disrupted other essential health services in Africa. This disruption has threatened the control of major high-burden diseases such as HIV, tuberculosis (TB), and malaria as well as the prevention of maternal and child mortality.
- While the 2020 WHO global reports for HIV, TB, and malaria show progress in control of these diseases in African countries, there are still significant gaps in meeting the global targets. Similarly, modeling studies predict that most African countries are unlikely to meet the Sustainable Development Goals (SDGs) 2030 targets for reductions in maternal and child mortality under the current rate of progress.
- Prediction models and emerging empirical data indicate that the implemented mitigation measures against COVID-19 such as travel restrictions and lockdowns as well as the repurposing of health resources and suspension of prevention programs such as immunizations will lead to an increase in new infections and deaths, significantly reversing the gains achieved in the control of these health challenges.
- A more comprehensive COVID-19 response that minimizes indirect deaths is therefore warranted in Africa. These include implementing WHO recommendations that limit contact with the clinic where possible, such as multimonths drug dispensing, self-testing, virtual platforms for case management, community- and home-based prevention, and care services such as home distribution of test kits, vaccines, treatment, and mosquito nets.
- This is in addition to ensuring effective implementation of COVID-19 infection prevention and control measures in healthcare facilities including providing healthcare workers with personal protective equipment and prioritizing them for COVID-19 immunization.
- There is also a need to incorporate aggressive recovery plans to reverse the lost gains in disease control efforts and put African countries back on course toward achieving the global targets. This includes leveraging on the wider COVID-19 response enablements such as the increased political will and global solidarity funding efforts to support a more comprehensive response that accounts for the indirect public health effects of the pandemic.

## Introduction

In 2020, the Severe Acute Respiratory Syndrome Coronavirus 2 (SARS-CoV-2) causing Coronavirus Disease 2019 (COVID-19) spread rapidly, resulting in a pandemic that is still ongoing in most countries. Africa’s response to the pandemic was largely decisive with various levels of implementation of nonpharmaceutical intervention measures [1]. At the beginning of the first wave of the pandemic in April 2020, 88% (43/49) of African countries had implemented stringent nonpharmaceutical intervention including some level of closure of the public transport system, staying-at-home measures, and travel restrictions, but these measures have since been relaxed in most countries [2]. The implemented public health and social measures have contributed to limiting the spread of COVID-19 in Africa with the number of new cases significantly dropping by the end of August 2020. However, a second wave has been reported in most African countries since September 2020. As of 27 April 2021, the continent had experienced a total of approximately 4.52 million cases and 120,420 deaths, which account for 3.1% of the global cases and 3.9% of the deaths [3].The drastic measures restricting social contact and movement of people, as well as the fear of visiting healthcare facilities, have, however, affected healthcare services for non-COVID conditions [4–6]. In addition to reallocation of resources such as healthcare personnel and diagnostic equipment, to effectively combat the pandemic, shortage of medical supplies arising from disruption in supply chain systems have further compounded the indirect impact of COVID-19 on other health conditions in Africa [4–6].

Experience from past epidemics shows that disruptions in healthcare systems result in a significant number of indirect deaths [7–9]. Modeling and empirical studies indicate that during the 2014 to 2016 Ebola epidemic in West Africa, more deaths were attributed to disruptions in malaria, tuberculosis (TB), HIV, maternal and child health (MCH) services than from Ebola infection [7–9]. Significant disruptions in healthcare services have also been observed and predicted in the current pandemic. Data from a national survey in South Africa in April 2020 showed that 13.2% of participants surveyed were unable to access medication for chronic diseases during the lockdown, suggesting that the implemented COVID-19 mitigation measures could significantly impact access to critical healthcare services [10]. A household survey in 18 countries by Partnership for Evidence-Based Response to COVID-19 (PERC) in Africa in August 2020 reported up to 44% delayed healthcare visits and up to 47% difficulties in obtaining medication [4]. In addition, excess non-COVID deaths of approximately 28,000 were reported in South Africa from early May to late October 2020, which may potentially be due to indirect effects of COVID-19 [11].

COVID-19 and the containment measures are also likely to impact the response to other epidemics. Between January and December 2020, an approximately 58 non-COVID-19 disease outbreaks were reported in Africa [12]. Responding to multiple epidemics could be a challenge especially with the strict lockdown measures and the repurposing of healthcare personnel and resources for COVID-19 response. Overall, COVID-19 and the implemented mitigation measures to contain it are likely to have a long-lasting impact that transcends the immediate effects posed by the virus. In this article, we describe the potential impact of COVID-19 on HIV, TB, malaria, MCH programs in Africa, the mitigation strategies to reduce this impact, and lessons learned to guide a more comprehensive and integrated response.

## The indirect impact of COVID-19 on HIV, TB, malaria, and maternal and child health programs

Africa bears a disproportionate burden of HIV, TB, and malaria as well as unacceptable levels of child and maternal mortality [13–17]. In 2019, African countries accounted for 67% of the global total of 38 million people living with HIV, 25% of the 10 million TB cases, and 94% of the 215 million malaria cases [14,15,17]. Altogether, the 3 infectious diseases accounted for approximately 1.2 million deaths in 2019, a number 10× higher than the current cumulative COVID-19 deaths reported in the continent since the beginning of the pandemic [14,15,17,18]. Significant progress has, however, been made to combat these diseases with projections to end the epidemics of HIV, TB, and malaria and significantly reduce child and maternal mortality set at 2030 as per the Sustainable Development Goals (SDGs) [13–17]. However, global reports indicate that countries are still offtrack in achieving the proposed targets with modeling studies predicting possible reversal in the already attained gains due to COVID-19 and its containment measures [13–17].

### HIV

The 2020 UNAIDS report showed that the interim target of having 73% of people living with HIV on treatment and virally suppressed was offtrack in Africa by 11.8 percentage points in 2019 [14]. The report further suggests that it is unlikely that the gap was met in 2020 citing also the potential disruptions from COVID-19 [14]. Modeling studies conducted in the early periods of the pandemic further predict a large increase in incidence and deaths attributed to disruptions in HIV prevention and treatment services. This includes an additional 440,000 to 736,000 HIV deaths attributed to a 6-month disruption in treatment access in 50% of the population of people living with HIV who are on treatment [19]. Overall, this reflects a 9-year rollback of progress in HIV control [19]. Similarly, a 3-month disruption in prevention of mother-to-child transmission of HIV services is projected to result in an increase in vertical transmissions of between 40% and 80%, which will significantly impede the goal of elimination of mother-to-child HIV transmissions among African countries [19].

Although empirical data confirming the model predictions are largely missing, a few surveys and studies seem to indicate significant disruptions in health services.

A survey by WHO conducted between April and June 2020 showed that 19 of 33 (58%) African countries had a potential risk of antiretroviral (ARV) treatment service disruption [5]. Moreover, 58% (7/12) of the countries reported having less than 3 months’ supply of ARVs in-country [5]. In addition, up to 14% of patients in Kenya [20] and 19% in Zimbabwe [21] had missed drug refills based on surveys conducted in April 2020, suggesting a potential risk of poor treatment outcomes including the emergence of HIV drug resistance. However, a recent survey by UNAIDS in October 2020 showed that there had only been mild disruptions in access to ARV treatment but significant declines in HIV testing and ART initiation based on data from 28 countries [22]. Similarly, a large study in South Africa comparing data prior and during the pandemic (collected between January 2018 and July 2020) reported mild disruptions in drug pickup but significant declines in HIV testing and treatment initiations [23]. Countries have also reported potential disruptions in other HIV services including testing, viral load monitoring, prevention services like voluntary medical male circumcision services, preexposure prophylaxis, early infant diagnosis for prevention of mother-to-child transmission, among others [5]. It is, however, worth noting that recent studies have reported improvements and recovery in HIV services with the relaxation of lockdown measures [22,23].

### Tuberculosis

WHO End TB strategy 2020 interim targets of reducing TB incidence by 20% and deaths by 35% from 2015 levels are also offtrack [15]. WHO global TB 2020 report showed that the continent had achieved only a 16% reduction in incidence and 19% reduction in deaths by 2019, suggesting the unlikelihood of the targets being achieved under the same progress rate [15].

Modeling studies commissioned by WHO in 2020 further predict a 13% increase in TB deaths arising from a 25% disruption in TB services due to COVID-19, over the course of 3 months [24].

Disruptions in TB services of up to 78% have been reported, based on survey data by The Global Fund to Fight AIDS, Tuberculosis, and Malaria (Global Fund) in 106 countries as of June 2020 [25]. Nigeria and South Africa, the 2 high-burden TB countries in Africa, have reported a 34% and 33% decrease in active TB case notification, respectively, at some point during the lockdown [26,27]. Missed drug refills have also been reported in other African countries [28]. Overall, the current measures taken to contain COVID-19 as well as repurposing of resources for the pandemic response are likely to limit access to treatment and diagnostic services potentially increasing new cases including drug-resistant TB and deaths.

### Malaria

The progress in attaining the malaria global targets has been dismal with the 2019 incidence rate being 225 per 1,000 population against the 2020 interim target of 139 per 1,000 population and mortality rate of 40.3 per 100,000 against the target of 29 per 100,000 at-risk population [17].

Modeling studies predict that a 25% decline in the distribution of insecticide-treated nets (ITNs) and access to artemisinin-based combination therapy (ACT) would lead to a 12% increase in malaria cases and a 35% increase in deaths [29]. However, a worst-case scenario of a 75% decrease in ITN distribution and ACT access is predicted to result in a 22% increase in incidence and doubling of malaria deaths to 769,000, reflecting mortality levels not observed in the last 2 decades. However, more recent data suggest that most countries in sub-Saharan Africa were still able to conduct the mass distribution of ITN, therefore minimizing this impact [17]. Nonetheless, even with this, disruption in access to ACTs by 10% to 50% is predicted to result in 19,000 to 100,00 additional deaths in Africa [17].

A survey conducted by the Global Fund in 106 countries indicated disruptions in up to 73% of malaria control services as of June 2020 [25]. Moreover, some countries experienced major delays in the mass distribution of ITNs (7 of 31) and indoor residual spraying (IRS) (8 of 31) in 2020 [17]. A resurgence in malaria cases after interruption of control strategies have been known to occur in the past. For example, in Zimbabwe, the incidence of malaria cases and deaths was shown to have doubled between February and April 2020 compared to the numbers reported at the same time point in 2019 [30].

### Maternal and child health

Globally, Africa accounts for the highest under-5 mortality, with 1 in 13 children dying before their fifth birthday [13]. Modeling studies further predict that unless current efforts are intensified, it is unlikely that 35 African countries would be able to meet the SDG target for reducing under-5 mortality rate [13]. In addition, under the current progress rate, the continent is likely to also miss the 2030 target for reduction in maternal mortality [16].

Recent modeling studies suggest that disruption in essential MCH services (family planning, antenatal care, childbirth care, postnatal care, early child vaccination, and early child preventive and curative services) by 39% to 52% and 50% wasting due to reduced access to food for 6 months will result in 557,000 under-5 child deaths in Africa [31]. Maternal mortality is also predicted to increase by 28,392 deaths in Africa owing to 6-month disruptions in access to skilled childbirth services [31].Overall, these models predict a significant rollback of progress in improving population health in the continent.

Disruptions in MCH services due to COVID-19 and its mitigation measures have also been reported in the continent. In Kenya, for example, in-hospital delivery rates dropped by 70% in April 2020 [32], while in Uganda, maternal mortality increased by 82% between January and March 2020 [33]. In addition, a recent WHO survey showed that up to 1.37 million children in Africa missed the TB, Bacille Calmette–Guérin (BCG) vaccine, while 1.32 million children missed the measles vaccine between January and August 2020 [34]. The scaling down or overall suspension of immunization services poses a risk for outbreaks of vaccine-preventable diseases [32,35]. In the 2013 to 2016 Ebola outbreak in Liberia, a 30% drop in measles immunization led to a 5-fold increase in measles incidence [35]. In Sierra Leone, antenatal care services reduced by 22%, facility deliveries by 9%, and postnatal care by 13%, which potentially may have led to the additional 3,600 indirect maternal and neonatal deaths and stillbirths, a number nearly equivalent to the direct deaths from Ebola [7]. In addition, a potential decline in quality of healthcare services or late access to care was associated with a 34% increase in maternal deaths and a 24% rise in stillbirths among those who had facility-based delivery [36].

## Comprehensive COVID-19 response with concomitant control of HIV, TB, malaria, and maternal and child deaths

With the potential for comparative high indirect deaths from the continent’s other major health threats, there is a need for a more comprehensive and balanced COVID-19 response strategy that minimizes its negative indirect impacts as discussed below.

### Innovative measures that ensure the continuation of essential healthcare services

At the beginning of the pandemic, WHO and other expert groups released guidelines for maintaining essential health services during the pandemic, which also included innovative measures that ensures the continuation of prevention, testing, and treatment services while minimizing contact with the clinic where possible, as summarized in Table 1 [5,15,37,38]. This includes multimonth dispensing for HIV and TB drugs, self-testing for HIV and reliance on community-based services for delivery of treatment, testing, and preventive services as well as using digital support systems such as telephone and text messaging for treatment support and surveillance [5,15,37,38]. Wide implementation of these strategies is critical as countries experience new waves of the pandemic. This is in addition to training healthcare workers including the community healthcare workers on infection prevention and control measures, as well as providing them with personal protective equipment and prioritizing them for COVID-19 immunization.

**Table 1:** Summary of indirect impact of COVID-19 and the recommended mitigation strategies to minimize its impact on HIV, TB, malaria, and MCH programs.

Disease control program	Impact from COVID-19	Mitigation strategies
HIV	Increase in HIV incidence and attrition, drug resistance, and deaths due to disruptions in access to prevention, treatment, and monitoring services as well as structural factors impacting treatment adherence	• Adapting and scaling up differentiated service delivery including multimonth ARV dispensing • Integrating HIV testing into COVID-19 community-based testing • Promote and prioritize self-testing to high-risk or vulnerable groups such as partners of people living with HIV and pregnant women • Adopting digital/virtual health service delivery platforms to support treatment initiation, psychosocial support, and adherence • Promoting home-based care for patients with advanced disease • Supporting community-based, home-based, and pharmacy-based distribution of prevention tools, self-test kits, ARVs as well as blood draws for viral load, and early infant diagnosis testing • Adopting digital platforms for surveillance and monitoring such as monitoring stock levels for timely distribution of commodities
TB	Increase in TB incidence, drug resistance, and deaths	• >1 month dispensing of anti-TB drugs for treatment and prevention • Adapting community-based model of care including delivering treatment and adherence such as direct observed treatment supported by family members or self-administered therapy coupled with increased literacy • Integrating TB diagnosis in COVID-19 testing strategies • Adopting virtual digital health technologies for patient care and support including VOT
Malaria	Increase in incidence and deaths	• Community-based testing, treatment, surveillance, and distribution of preventive measures including seasonal malaria chemotherapy • Innovative distribution channels of prevention campaigns like use of motorcycles for door-to-door distribution of ITNs so as to ensure physical distancing and minimize mass gatherings common with the convention mass campaigns • Providing frontline healthcare workers including those involved in IRS with COVID-19 prevention equipment and prioritizing them for COVID-19 vaccination • Continuing prevention campaigns including chemoprevention services, ITN distribution, and IRS while ensuring COVID-19 infection, prevention, and control measures
MCH	Increase in deaths and disability	• Using community outreach platforms for antenatal and postnatal care and immunization services • Leveraging any hospital visits for mother and child to provide other essential services like nutrition support and immunization for children and antenatal and postnatal care for mothers • Ensuring continuity of MCH services while strengthening infection, prevention, and control measures • Intensive catch-up campaigns • Spacing out maternal and child visits to avoid overcrowding in hospitals • Strengthening data systems to facilitate identification of children missing immunization schedules who can be prioritized during catch-up campaigns • Use of digital technologies for counseling, health promotion, and screening for danger signs • Targeted outreach services for both antenatal and postnatal care

ARV, antiretroviral; COVID-19, Coronavirus Disease 2019; IRS, indoor residual spraying; ITN, insecticide-treated net; MCH, maternal and child health; TB, tuberculosis; VOT, video-observed therapy.

Overall, a dual-track health system that accommodates reinstatement of essential health services and protects healthcare workers, while at the same time supporting an effective COVID-19 response, is needed as immediate measures to minimize indirect effects of the pandemic.

In addition, catch-up health campaigns including immunization and malaria preventive interventions, active case finding for TB, addressing wasting in children as well as active tracking, tracing, and reengagement in care for TB and HIV patients lost to follow-up, coupled with appropriate message campaigns, need to be urgently implemented.

There is also an overall need for better comunication strategies with simplified and clear messaging to ensure that both COVID-19 infection prevention and control measures as well as strategies to mitigate its indirect effects are implemented and adhered to. Moreover, it is critical for government leaders to work together with public health experts to avoid conflicting messaging and also support the implementation of these measures.

### Leveraging novel innovations developed for COVID-19 control

There is a need to leverage positive innovations developed to combat COVID-19 to support other disease control programs as well as MCH. At the continental level, Africa CDC jointly with partners has promoted various initiatives to support COVID-19 response in Africa, including digital health platforms for surveillance, building up and facilitating the use of genomic-based pathogen surveillance, building new partnerships to facilitate access to diagnostics and vaccines, and promoting pooled procurement of medical supplies as well as in-continent manufacturing of diagnostics to correct supply side distortions experienced during the pandemic (Table 2) [39–41].

**Table 2:** Summary of best practices, innovations, and other enablers developed to support COVID-19 response that could be leveraged to support other disease control programs.

Best practices and technologies	Application to other disease control programs
Integrated diagnostics/surveillance	Leveraging on COVID-19 contact tracing and ongoing serological surveys and other testing campaigns to also support the screening of HIV, TB, and malaria. Overall integrated diagnostics approach can be used since COVID-19 testing leverages also on both HIV and TB testing equipment
Integrated real-time health surveillance systems	Extending real-time digital surveillance systems to collects strategic information on other health conditions including, but not limited to, facility-based data on retention for HIV and TB, malaria incidence and treatment, and childhood immunizations as well as excess indirect deaths. Overall real-time surveillance for the aforementioned health programs needs to be done so as to provide evidence for reallocation of resources during COVID-19 based on areas of greatest need
Community- and home-based care service delivery	Leveraging on CHW and rapid responders deployed to support community-based COVID-19 response to also support other disease control programs in such activities as distribution of test kits, ITNs, among others. CHWs should, however, be capacitated with protective personal equipment, training on infection, prevention, and control measures, as well as be prioritized for COVID-19 vaccination
Innovative training and capacity building methods	Virtual training platforms for sharing of best practices in COVID-19 response can be leveraged to support widescale adoption of the proposed mitigation strategies across malaria, HIV, TB, and MCH programs. In addition, virtual training systems can be used to train and deploy a mass of community health volunteers to support other health programs
Local manufacturing capacities	Several countries like Senegal and Morocco have been able to establish in-country production line for diagnostics, which can be leveraged to support manufacturing of other tests including rapid diagnostic tests for malaria and HIV
Pooled procurement system for medical commodities	Leveraging on the African Union continental pooled procurement system to ensure adequate supply of medical commodities for the other health programs. The pooled procurement system could help address the supply chain challenges being experienced in the continent for other medical commodities as well as ensure affordable pricing through bulk purchasing and joint negotiations
Expanded genomic-based surveillance	Leveraging the COVID-19 expanded genomic capacity in the continent to combat endemic infectious diseases, e.g., assessing resistance for anti-TB drugs, ARVs, and malaria drugs as well as enhancing surveillance for potential outbreaks of vaccine preventable diseases in children. In addition, genomics could be used to promote network surveillance for TB and HIV
Strategic partnership, collaborative efforts, and political leadership	The unprecedented political will and global efforts to support COVID-19 response can be leveraged to support the other disease control programs and accelerate the progress toward attaining the targets for HIV, TB, malaria, under-5, and maternal mortality rates. Overall, there is a need to change the narrative for COVID-19 response to include the need to minimize indirect deaths. Similarly, there is need for global advocacy coupled with collective and rapid resource mobilization to support control efforts for these other health threats during and after COVID-19

ARV, antiretroviral; CHW, community health worker; COVID-19, Coronavirus Disease 2019; ITN, insecticide-treated net; MCH, maternal and child health; TB, tuberculosis.

Other partners like the African Society of Laboratory Medicine have also been instrumental in facilitating the sharing of best practices as well as innovative online training through digital platforms, which can further be expanded to support other disease control and health programs [42]. In addition, Africa CDC has been instrumental in coordinating and promoting a strong political will, joint continental collaborations, central coordination efforts, open data sharing systems, strengthening of regional integration, and a wider collaboration for disease control efforts. Overall, these strategies need to be further expanded to support malaria, TB, HIV, and MCH programs during and after the pandemic.

In addition, as others have also recommended there is a need to integrate COVID-19 surveillance and response with the other major health threats in the continent [43]. This includes wide adoption of the various tracking tools developed to monitor disruptions of other essential health services such as WHO recommended indicators for monitoring the effects of COVID-19 on essential services [44] and integrating the data into the wider COVID-19 response. Moreover, experts from the different disease control programs and MCH also need to be included in the COVID-19 response task forces. In addition, there is a need to strengthen health data systems to facilitate reporting of important metrics such as excess deaths in addition to COVID-19 deaths in order to guide reprioritization of resources and review of mitigation strategies. The gaps in data systems are also a major challenge in Africa and impede accurate determination of the indirect effects of COVID-19. This continues to highlight need to accelerate implementation of better data systems leveraging on existing digital platforms. Overall, a more integrated response coupled with better data systems will enable efficient forecasting and use of the limited diagnostic capacity, personal protective equipment, and efficient deployment of healthcare workers including community healthcare services as well as financial resources.

### Accelerating the implementation of universal health coverage

The indirect socioeconomic effects of COVID-19 have further worsened existing health disparities, significantly affecting the ability of the most-at-need populations to access quality healthcare. As such, there is a critical need to accelerate the implementation of the 2030 universal health coverage (UHC) goal to cushion this population. Moreover, early findings indicate better COVID-19 pandemic responses in countries with good UHC such as Taiwan, Vietnam, and Singapore [45], suggesting the pivotal role of UHC in epidemic preparedness and response. It is also worth noting that out-of-pocket costs for COVID-19 treatment and isolation have proved to be expensive in the continent and out of reach for most Africans. As such, accelerated implementation of UHC is critical to support both COVID-19 and non-COVID conditions.

Despite the commitment made by most African countries including the wide integration of UHC into national health strategies, the actual progress has been dismal, and this may have affected the ability to mount an effective response against COVID-19 as well as maintain routine healthcare services. A 2021 report assessing the state of UHC in Africa shows that only 48% of the population receives the healthcare services that they need, while up to 97 million people (8.2%) experience catastrophic healthcare costs [46]. It is further estimated that 15 million people are pushed into extreme poverty every year due to the catastrophic out-of-pocket healthcare costs [46]. Moreover, domestic financing is still a challenge with only 4 countries reporting having met the Abuja target of having 15% of total government expenditure spent on health [47]. The challenge in access to COVID-19 quality healthcare as well as the need for control and eradication of endemic diseases highlights the need for accelerated implementation of UHC. As highlighted by WHO and others, including the present authors, there is a need to implement UHC and global health security enablers, including increasing domestic financing for health systems strengthening from such sources as government tax revenues and insurance; close monitoring and reporting of UHC implementation metrics; strengthening the public healthcare workforce; strengthening public health institutions including capacities for integrated disease surveillance such as laboratories, emergency operation centers, and innovative information systems as well as primary healthcare systems; creating mechanisms for a strong community engagement to foster accountability; strengthening systems for quality data generation, use, and sharing; building capacity for local manufacturing of vaccines, therapeutics, and diagnostics; and establishing mechanisms to ensure that efforts by donors and partners are better coordinated and aligned to continental and countries’ health aspirations [47–50]. Concurrent with accelerating UHC implementation, setting up infrastructure and systems to measure and report on the indirect effects of health and humanitarian emergencies on other health services, plus intra and post-emergency recovery, at country, regional, and continental level would be very important.

### Financing health recovery plans

Resource mobilization to support a more comprehensive and integrated strategy may however be challenging for most African countries due to shrinking economies, weakened local currencies, and potential budget cuts. Some countries have in fact already adjusted to COVID-19 by cutting health budgets [51,52]. As such, there is a need for additional global support. The level of global solidarity to address the current pandemic, although falling short especially in ensuring global access to COVID-19 vaccines, has been unprecedented in our age, and the same is needed to support health recovery efforts and ensure that global targets for improving public health in African countries remain on track. Overall, a more comprehensive continental strategy that accounts for the indirect effects of COVID-19 with subsequent funding mobilization is needed. Nonconventional funding strategies employed in COVID-19 including donations from philanthropists, private companies, and individual government–government support as well as debt relief for health will also be critical in supporting this strategy. In addition, accelerating implementation of UHC may help increase domestic funding in the wake of diminishing donor funding.

## Conclusions

The COVID-19 pandemic presents multifaceted challenges to many healthcare programs: from reduced access to healthcare services including testing, treatment, and care support services, to structural impacts on drug stockouts, resource shortages, and malnutrition. While every effort must be put in place to contain the pandemic, neglecting endemic diseases and MCH is likely to lead to a greater loss in population health in African countries with comparatively more indirect deaths. An integrated pandemic response that ensures continuity of endemic disease control efforts and MCH services and catch-up campaigns for essential disease prevention and control activities is urgently needed. Post-pandemic recovery plans must also include structural changes in public health systems to ensure rebuilding resilient systems that are capable of supporting effective epidemic response while at the same time ensuring continuity of essential health services. Sustained international solidarity as observed during COVID-19 is required to support an integrated COVID-19 response that ensures the continent remains on course toward achieving the global targets for HIV, TB, and malaria control and MCH mortality reductions.

### Disclaimers

The authors alone are responsible for the views expressed in this publication, and they do not necessarily represent the decisions or policies of the Africa CDC or ASLM.

## Abbreviations

ACT: artemisinin-based combination therapy
ARV: antiretroviral
BCG: Bacille Calmette–Guérin
COVID-19: Coronavirus Disease 2019
IRS: indoor residual spraying
ITN: insecticide-treated net
MCH: maternal and child health
PERC: Partnership for Evidence-Based Response to COVID-19
SARS-CoV-2: Severe Acute Respiratory Syndrome Coronavirus 2
SDG: Sustainable Development Goal
TB: tuberculosis
UHC: universal health coverage
